# Impacts of educational debt on physical therapist employment trends

**DOI:** 10.1186/s12909-023-04454-3

**Published:** 2023-06-20

**Authors:** Tara Dickson, Edward P. Mulligan, Eric J Hegedus

**Affiliations:** Department of Rehabilitation Sciences, Tufts University School of Medicine, 101 E Washington Street, Suite 950, Phoenix, AZ 85004 USA

**Keywords:** Retirement, Work volume, Income, Human capital, Job satisfaction

## Abstract

**Background:**

Newly graduated physical therapists have high amounts of educational debt.

Educational debt may negatively affect job satisfaction, aspirations for professional development, and choice of workplace setting. Research has not shown this association directly, yet it is conceptually supported by the Labor-Search Model. The purpose of this study was to understand the role that educational debt has on additional factors related to job choice in the Labor-Search Model.

**Methods:**

Retrospective data were captured through the Virginia Longitudinal Data System (VLDS) for 12,594 licensed physical therapists within the Commonwealth of Virginia from 2014 to 2020. A fixed effects panel analysis, with inflation-adjusted educational debt as the variable of interest, was conducted to assess whether patterns of professional certifications, volume of work, workplace setting, and job satisfaction were related to educational debt.

**Results:**

Educational debt was positively correlated with higher professional degrees (*p* = 0.009), the number of hours worked per week (*p* = 0.049), and expected number of years until retirement (*p* = 0.013). Job satisfaction was statistically significant (*p* = 0.042) and negatively correlated with educational debt.

**Conclusions:**

Those with higher educational debt appear to have the habit of working more hours per week and have a longer time horizon until retirement. Newly licensed physical therapists with higher amounts of educational debt are more likely to experience this trend. Income and job satisfaction demonstrated an interaction effect on educational debt, such that those with lower levels of income had a stronger, negative relationship between their debt and job satisfaction, as compared to those with higher income.

## Introduction

Multiple factors affect the physical therapist workforce including supply of physical therapists, demand for services, job satisfaction, and educational debt. However, the influence of these factors is hotly debated in the United States (US) [1, 2], and early career physical therapists (and, ultimately, their patients) will bear the cost of these workforce conditions. In 2022, the US Bureau of Labor Statistics projected 21% growth of physical therapist jobs by 2030 [3]. Conversely, according to a 2020 American Physical Therapy Association (APTA) analysis of the physical therapist workforce, the supply of physical therapists is expected to exceed the demand for physical therapist services in 2024 by 10,000 across the country, with a surplus of 25,000 by 2030 [4].

The contradiction in supply and demand curves is due to several factors. First, an increase in the rate of development of new physical therapist programs has increased supply [5]. Second, the COVID-19 pandemic has had a mixed effect on the workforce resulting in temporary furloughs and career changes of some physical therapists [6], while patient complications, such as Long COVID, may increase demand for rehabilitation services [7, 8]. Third, there is no accounting for physical therapists who have retired or left the workforce, which would decrease supply [1].

Beyond supply and demand factors, educational debt is impacting the physical therapist workforce. The US Bureau of Labor Statistics reported a 63% rise in US college tuition and fees, compared with a 21% rise in consumer price index, from 2006 to 2016 [9]. A national survey of student physical therapists found that DPT students attending public institutions had over $103,000 in total student debt, while those attending private institutions had over an average of $138,000 in total student loan debt [10]. Importantly, those that have student debt over $200,000 may not be able to meet their recommended loan repayment benchmarks [11]. Like the trends in the pharmacy [12, 13] and physician associate [14] professions, the physical therapy profession has realized gains in student educational debt while salaries remain relatively flat [15]. Further, the physical therapist profession has far less earning potential than other healthcare professions, such as medicine and pharmacy [15]. Thus, the income to debt ratio for health professionals, and particularly physical therapists [16], continues to decline.

Educational debt is known to affect the post-professional aspirations of physical therapists [16, 17], affects life satisfaction [18], limits one’s ability to save for the future [19], and ultimately influences choice of work setting [20, 21]. It is, therefore, imperative that the profession be aware of any impending storm clouds that may impact the physical therapist workforce as it relates to educational debt [2]. A systematic review of medical student debt found a significant association between educational debt and the pursuit of a higher paying specialty [22]. Another study found a significant correlation between the pursuit of residency specialty selection based on earning potential [23]. These choices could affect long-term job satisfaction or the attainment of additional post-professional certifications or credentials. The present study seeks to fill a gap in the current literature to determine if higher educational debt is associated with seeking higher salaries, working more hours, differences in job satisfaction, and how these co-variates may relate to satisfaction with a physical therapist’s employment situation.

### Conceptual framework

This research is grounded in the Labor-Search Model and Human Capital Theory. Human Capital Theory predicts that individuals will invest in higher education to build their own marketable skills, which are then used to solidify economic success [24–26]. This results in a competitive, “meritocratic” environment in the US [27]. Educational training, particularly the pursuit of higher educational degrees, professional training, and specialties, is a major way in which one invests in their own human capital to ensure a financially secure future [26]. For this reason, high tuition is generally not a deterrent for students attending college [28]. Rather, students are likely pursuing professional degrees in attempt to secure their economic futures, in combination with a passion for that profession.

The pursuit of professional healthcare degrees has been increasingly resulting in high educational debt for graduates [10–15]. The results of this debt will are seen within the professional workforce, but individual responses to educational debt will manifest in various ways as professionals seek to raise their own human capital [26, 29]. The Labor-Search Model allows us to predict the employment behaviors of health professionals, and specifically physical therapists, who have educational debt [30].

**Fig. 1:**
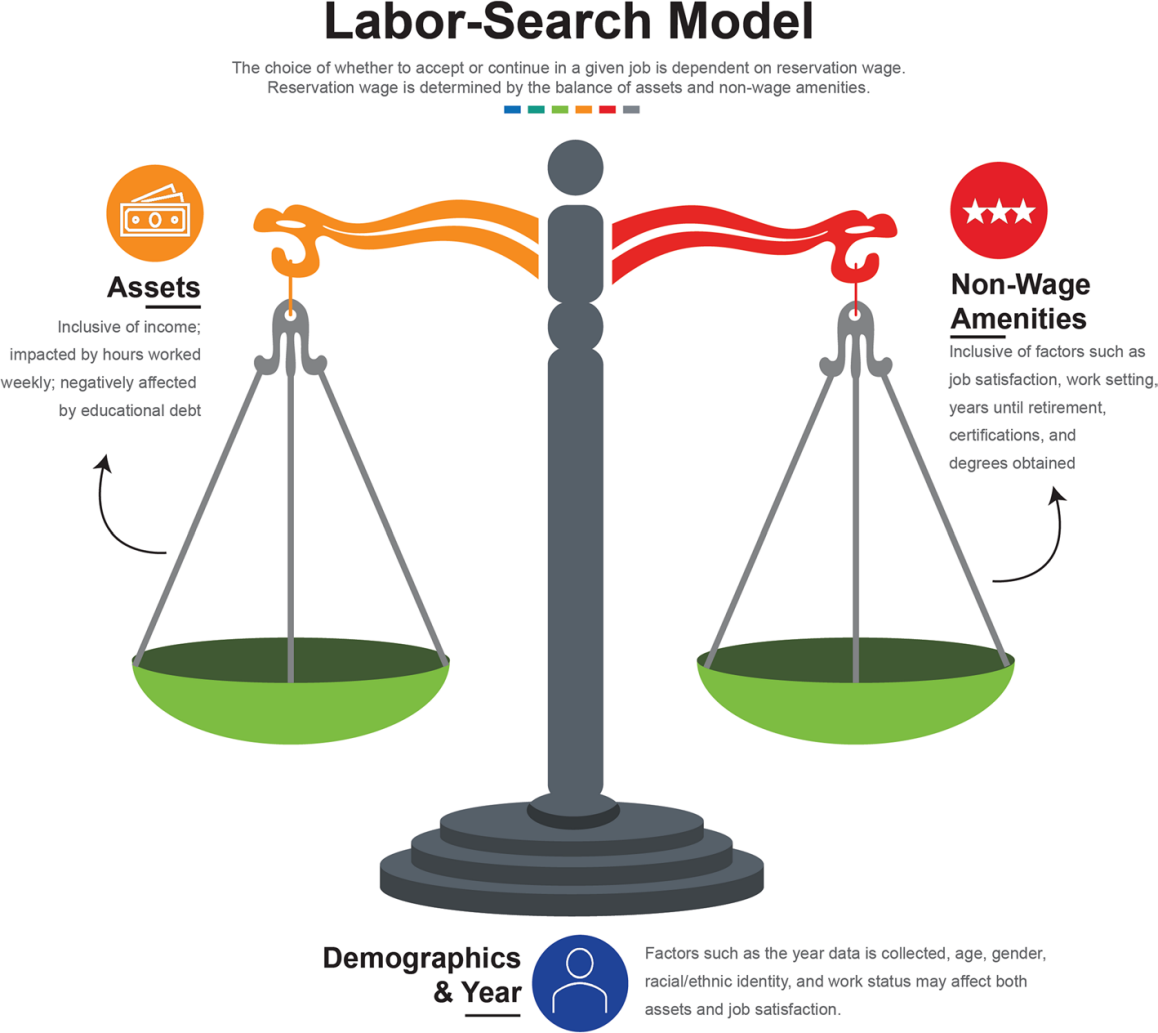
The Labor-Search Model as conceptualized for the present study. Adapted based on the work of M Luo & S Mongey [19]

In the Labor-Search Model, the choice of whether to obtain or keep a job (known as “job choice”) is dependent on whether that job promises a high enough “reservation wage” [30]. Reservation wages include a balance of both assets and non-wage amenities [30]. In the model presented in Fig. 1, assets include both annual income and the number of hours an employee chooses to work weekly. Additional factors, such as employee age, gender, race, ethnicity, and work status may also affect the balance of reservation wages, and thus they are included in the Labor-Search Model.

Non-wage amenities can include perceived benefits of working in a position [30]. That can include factors such as job satisfaction, work setting, and the number of years until retirement; as well as measures of professional development, like degrees and specialty certifications obtained. Research demonstrates that educational debt affects the choices one makes by influencing graduates to choose higher-paying positions over positions that may provide more “satisfying,” but lower-paying public interest positions [21]. Additional cited methods of increasing reservation wages in the literature are through working more hours [31], pursuing specialty training [32], or working in a higher-paying setting [33, 34].

The Labor-Search Model states that if an employment position provides a high enough salary, a prospective employee will take that position, even if perceived job satisfaction or non-wage amenities are lower because the salary is high enough to raise the “reservation wage” to the allowable threshold [30]. The converse is also true—some prospective employees will take a lower-salary position if it promises a high enough job satisfaction and work-life balance. The Labor-Search Model is particularly useful for us because it also predicts that educational debt will negatively impact the “assets” piece of the reservation wage [30]. In other words, if a prospective employee has high educational debt, that employee will typically look for a position that provides much higher wages and be more willing to sacrifice job satisfaction and work-life balance for financial security.

We use the Labor-Search Model as illustrated in Fig. 1 to observe the relationships between educational debt non-wage amenities within the physical therapist workforce. The purpose of this study is to determine whether there is an association between educational debt and physical therapist work setting, work hours, job satisfaction, and attainment of post-professional certifications.

## Methods

We obtained retrospective data through the Virginia Longitudinal Data System (VLDS) [35]. VLDS is a collaborative effort that securely links data from multiple agencies within Virginia to allow for longitudinal analysis of educational and workforce data within the state [35]. While we primarily requested data generated through the Virginia Department of Health Professions (VDHP), some data were collected through the Virginia Department of Education (VDOE). The VDHP provides voluntary surveys to licensed physical therapists in the Commonwealth of Virginia (among other health professionals) through their license renewal process [36]. Data on physical therapists are collected every two years. Of note, survey data are not consistent with a census of all licensed physical therapists in the state because licensees may choose to not complete the survey. However, response rates generally range from 75 to 90% of those renewing a license [36]. A full description of survey methodology is available on the VDHP Health Workforce Data Center website [37]. De-identified data were provided to researchers on this project using random numbers to identify each person’s data over time in a way that did not reveal any one person’s protected information or identity. Tufts University IRB exempted this study due to it being de-identified, retrospective data.

Data on the physical therapist workforce were available for the years 2014, 2016, 2018, and 2020 at the time of request. Because we were interested in how educational debt was associated with both assets and non-wage amenities, the predictor variable of interest was the dollar amount of profession-related educational debt still owed by each respondent, termed Educational Debt [37]. Of note, this variable excluded educational debt that was related to the physical therapists’ pre-professional degree(s).

We included several co-variates that could reasonably be attributed to either assets or non-wage amenities. Variables representing assets included income and number of hours worked per week. Non-wage amenities included the following variables: job satisfaction, primary work setting (categorical variable), expected number of years until retirement, number of ABPTS or other profession-related certifications, highest earned professional degree (categorical variable), and job satisfaction. Job satisfaction, an ordinal variable, was rated on a four-point Likert scale, asking the rater’s level of satisfaction to their current employment situation (1 = Very Dissatisfied, 4 = Very Satisfied) [37].

We also included several demographic variables that might affect job choice, as predicted by the Labor-Search Model: age (divided into ordinal categories of 5-year increments), gender (dichotomous variable as defined by VDHP), self-reported race/ethnicity category (as defined by VDHP), work status (i.e., employed, involuntarily unemployed, retired), and year. The year data were collected was added to the model because major historical events (such as the COVID-19 pandemic, for example) can impact yearly employment patterns.

Educational debt and income were adjusted for inflation using the Consumer Price Index from January 2022 [38]. The educational debt and income variables were logged to satisfy the assumption of a normal distribution and to provide for ease of interpretation of the results. Dollar figures for educational debt and income presented in the Results section of this paper include inverse transformations of the logged values for ease of interpretation.

Though additional variables were considered at the time of data request, we considered the amount of missing data and the overlapping scope of the variables when choosing the final variables for the analysis. For example, there were several available variables in the dataset that considered the volume of work a physical therapist completed: number of work locations, average hours worked in a week, total number of hours worked per year, and number of full-time equivalents. The variable “average hours worked in a week” was chosen because it had the least missing data, with 13.8% of data missing.

### Statistical analysis

Our panel of longitudinal data allowed us to implement a richer analysis than using a cross-section of data from any one year. We decided to run both: (1) a fixed effects and (2) a random effects model in a similar manner to prior studies that have used retrospective panel data [39, 40]. Fixed and random effects models are beneficial for analyzing panel data because they include both within- and between- effects [41], as individuals’ data is included over time. Another reason for our choice of running both fixed effects and random effects models is that they allow for unbalanced data. Data is unbalanced when there is not an entry for each person in the model for all years studied [41]. Physical therapists were likely to enter and exit the profession throughout the study period. Therefore, it was important that our method of data analysis allowed for flow of individuals in and out of the workforce.

Fixed effects models are particularly beneficial because they greatly temper the concern of omitted variable bias. Variables that were not measured, but that remain consistent among a given person during the study period, are eliminated as possible confounders because those unmeasured variables are time-invariant [41, 42]. This is because fixed effects models assume that effects among individuals are fixed, or unchanging, and that they are correlated with the co-variates in the model. Often, this is too strong of an assumption for the data [41, 42].

Random effects models, on the other hand, allow for each person to have their own “individual-specific effect,” and the models assume that the individual-specific effect is uncorrelated with the co-variates in the model. At times, the random effects model provides less consistent estimates than the fixed effects model [41, 42]. Thus, it is important to run both models and then conduct a Hausman specification test to determine which model more appropriately fits the data. The null hypothesis of the Hausman test is that the co-variates in the model are not correlated with person-specific error [43]. If *p*< 0.05 for the Hausman test, then the estimates from the fixed effects model are preferred over the random effects model [43].

Data were analyzed using Stata 17 statistical software [44]. We planned to utilize listwise deletion for missing data (in which all observations for an individual at a given point in time are deleted if data from at least one variable is missing) [45], unless more than 15% of the data for any one variable were missing, in which case we would compare the results of the analysis using listwise deletion with the analysis utilizing multiple imputation. If the results of the multiple imputation were similar to that of the listwise deletion, then estimates from the model that used listwise deletion would be preferred, as it would avoid false-positive results [41].

Because fixed-effects models eliminate confounding effect of time-invariant variables, it is not uncommon for model results to eliminate some variables that do not vary over time [41]. Several of our included demographic variables, including gender and race/ethnicity, are unlikely to greatly change over time. In these cases, the eliminated variables were assessed through descriptive statistics to detect a possible influence on the variable of interest.

Several conditions must be met prior to analyzing the results of fixed and random effects models. Among these are joint significance of the covariates, serial correlations within the time series data, and multicollinearity [41]. An F-test for joint significance was conducted in both the random and fixed effects models. The xtserial command in Stata was used to test for serial correlations, and multicollinearity was tested using the rmcoll command in Stata for the fixed and random effects models [46].

Once a model—either the fixed or random effects model—was determined to be preferrable due to providing more consistent and efficient estimates, we ran further analyses to refine the estimates. First, we checked for non-linear relationships by squaring the linear, statistically significant terms, adding the squared term back into the model, and conducting an F-test for joint significance. If the F-test found that *p* < 0.05, it was determined that the estimates for that term were more precise with the non-linear term added. We also checked for interaction effects post-hoc.

Coefficients in a fixed effects model represent how much educational debt is predicted to rise based on a one-unit change in the co-variate. However, due to the non-linear nature of these relationships, we will rely on graphical prediction models to evaluate the relationships between educational debt and the statistically significant co-variates.

## Results

The VLDS data contained 26,984 observations, which included 12,594 unique individuals’ data. Less than 15% of the data were missing except for three variables: (1) the annual income variable, of which 21% of data were missing, (2) expected years until retirement, of which 24% of data were missing, and (3) gender, of which 39% of data were missing. Upon further analysis, all missing income data was from the year 2014. No additional patterns were noted among the missing variables.

Descriptive statistics for the included variables are listed in Tables 1 and 2. Of note, of the 26,984 observations, 13,411 reported no current profession-related educational debt at the time of completing the survey, while 13,573 did report educational debt related to their profession. For those without educational debt, the log-transformed educational debt variable was set to $0. The median amount of debt owed by those in the 25–29 age group was $81,472.97, while the median amount of debt owed by those in the 40–44 age group and above was $0. The mean number of hours worked per week was 42.3 (standard deviation of 6) for those in the 25–29 age group and 37.4 (standard deviation of 13.6) for those in the 40–44 age group. This data suggests that early career physical therapists are working about five additional hours per week, on average, when compared to those 15 years older.

**Table 1:** Descriptive statistics of included quantitative variables included in the labor-search model

	n	25th Percentile	Median	75th Percentile	Range
Educational Debt	25,714	$0.00	$0.00	$39,187.20	$0 - $148,450.20
Age	26,984	32	42	52	27–62
Certifications	26,984	0	0	1	0–14
Income	21,280	$1,170.24	$76,142.03	$99,570.34	$0 - $146,427.00
Hours Worked Per Week	23,260	37	42	47	7–72
Expected Years until Retirement	20,484	15	25	35	2–60
Job Satisfaction (1 = very dissatisfied, 4 = very satisfied)	23,864	3	4	4	1–4

**Table 2:** Educational debt by demographic characteristic

	n	Mean Educational Debt	25th Percentile	Median	75th Percentile
Primary Work Setting					
Home Health	3,287	$17,508.40	$0.00	$0.00	$15,000.00
Skilled Nursing	1,772	$25,374.96	$0.00	$0.00	$45,000.00
Rehabilitation Facility	3,106	$37,131.95	$0.00	$999.00	$75,000.00
Hospital, Inpatient	2,215	$28,503.39	$0.00	$0.00	$55,000.00
Hospital, Outpatient	2,430	$30,959.85	$0.00	$0.00	$55,000.00
Private Practice, Solo	1,515	$25,458.14	$0.00	$0.00	$45,000.00
Private Practice, Group	3,540	$32,108.24	$0.00	$0.00	$55,000.00
Academic Institution	656	$15,406.12	$0.00	$0.00	$5,000.00
Gender					
Male	4,281	$32,766.89	$0.00	$1,082.55	$59,746.84
Female	11,916	$29,183.70	$0.00	$0.00	$48,883.78
Work Status					
Employed	23,737	$26,215.91	$0.00	$0.00	$50,383.54
Voluntarily Unemployed	532	$14,316.31	$0.00	$542.61	$1,186.41
Involuntarily Unemployed	117	$22,315.51	$0.00	$1,085.22	$16,294.59
Retired	160	$797.54	$0.00	$0.00	$1,085.22
Professional Degree					
Baccalaureate Degree	5,432	$1,082.16	$0.00	$0.00	$0.00
Master’s Degree	5,471	$9,903.20	$0.00	$0.00	$1,186.41
Doctorate Degree	14,115	$45,242.47	$0.00	$5,857.08	$92,336.03
Age Group					
25–29	2,693	$72,974.11	$1,085.22	$81,472.97	$135,788.30
30–34	4,664	$55,642.31	$0.00	$39,187.20	$114,062.20
35–39	3,961	$33,025.86	$0.00	$1,186.41	$59,746.84
40–44	3,591	$17,686.03	$0.00	$0.00	$27,157.66
45–49	3,274	$8,934.26	$0.00	$0.00	$1,170.24
50–54	2,733	$4,691.68	$0.00	$0.00	$1,085.22
55–59	2,200	$3,092.39	$0.00	$0.00	$0.00
60–64	2,598	$1,210.48	$0.00	$0.00	$0.00

Fixed and random effects models using educational debt as the dependent variable were created for those who reported educational debt, using both listwise deletion and multiple imputation. The same variables were statistically significant in both the models using listwise deletion and multiple imputation, so it was decided to use the models that relied on listwise deletion. An F-test of joint significance indicated that it was appropriate to analyze the results of the models (*p* < 0.001 in each case), and the test for serial correlations indicated that cluster-robust standard errors were not needed as a safeguard against false-positive results. Multi-collinearity was not present within either fixed or random-effects models. The Hausman specification test (*p* < 0.001) indicated that the fixed effects model provided more consistent and efficient estimates as compared to the random effects model. Therefore, we analyzed the results of the fixed effects model.

### Model results

After performing listwise deletion and eliminating individual records for which any study variables were missing, the fixed effects model included 5,141 observations from 3,516 individuals. Results of the fixed effects model are listed in Table 3, which includes the set of covariates included in the model. Gender and work status were both omitted in the model due to having little variability. Of note, of the 117 observations that were “involuntarily unemployed” in the sample, 81 (69.2%) occurred in 2020. The race/ethnicity categorical variable was not omitted, likely due to there being some variance among individuals represented within the dataset.

**Table 3:** Results of the fixed effects model with educational debt as the dependent variable (*n* = 5,141)

Variable	Coefficient	Std. err.	*p*-value	95% Confidence Interval	
				Lower Bound	Upper Bound
Age	-0.010	0.01	0.14	-0.02	0.00
Gender	[omitted]				
Professional Degree	0.279	0.11	0.01*	0.07	0.49
Professional Degree^a^	0.229	0.19	0.23**	-0.14	0.60
Interaction of Age and Professional Degree	-0.017	0.02	0.30**	-0.05	0.02
Number of Certifications	0.039	0.02	0.06	0.00	0.08
Ln(Income)	-0.004	0.01	0.60	-0.02	0.01
Hours Worked Per Week	0.004	0.00	0.049*	0.00	0.01
Hours Worked Per Week^a^	-0.0001	0.00	0.20**	0.00	0.00
Expected Years Until Retirement	0.006	0.00	0.01*	0.00	0.01
Expected Years Until Retirement^a^	-0.0002	0.00	0.27**	0.00	0.00
Job Satisfaction	0.048	0.02	0.04*	0.00	0.10
Job Satisfaction^a^	-0.013	0.03	0.63**	-0.07	-0.04
Interaction of Age and Job Satisfaction	0.005	0.01	0.27**	-0.004	0.015
Interaction of Income and Job Satisfaction	-1.259	1.79	0.48**	-4.76	2.22
Work Status	[omitted]				
Primary Work Setting, compared with Home Health					
Skilled Nursing	0.094	0.09	0.29	-0.08	0.27
Rehabilitation Facility	0.119	0.08	0.12	-0.03	0.27
Hospital, Inpatient	0.006	0.09	0.95	-0.17	0.18
Hospital, Outpatient	0.023	0.08	0.77	-0.13	0.18
Private Practice, Solo	0.026	0.08	0.75	-0.14	0.19
Private Practice, Group	0.092	0.08	0.23	-0.06	0.24
Academic Institution	-0.043	0.14	0.75	-0.31	0.23
Race or Ethnic Category, compared with American Indian or Alaska Native					
Asian	0.059	0.51	0.91	-0.95	1.07
Black/African American	0.030	0.53	0.96	-1.01	1.07
Hispanic	0.011	0.51	0.98	-0.98	1.00
Native Hawaiian or Other Pacific Islander	-0.253	0.62	0.68	-1.46	0.96
Some Other Race	0.244	0.50	0.63	-0.74	1.22
Two or More Races	-0.029	0.43	0.95	-0.87	0.81
White	0.178	0.46	0.70	-0.73	1.09
Year Surveyed, compared with 2016					
2018	-0.186	0.03	< 0.001*	-0.25	-0.13
2020	-0.403	0.03	< 0.001*	-0.47	-0.34

^a^Indicates a squared, nonlinear covariate

*Indicates statistical significance at *p* < 0.05

^**^Indicates that the F-test for joint significance for the interaction term was statistically significant with *p* < 0.05

There were four statistically significant findings among the variables studied in relation to educational debt: professional degree, hours worked per week, expected number of years until retirement, and job satisfaction. We tested for non-linear relationships for each of the statistically significant variables by creating squared terms for each. F-tests for joint significance were run, and in each case, *p* < 0.001, indicating that the estimates from the fixed effects model were more precise with the nonlinear, squared terms included. Thus, prediction models presented are representative of the fixed effects model with the four squared terms for professional degree, hours worked per week, expected number of years until retirement, and job satisfaction included.

#### Professional degree

**Fig. 2:**
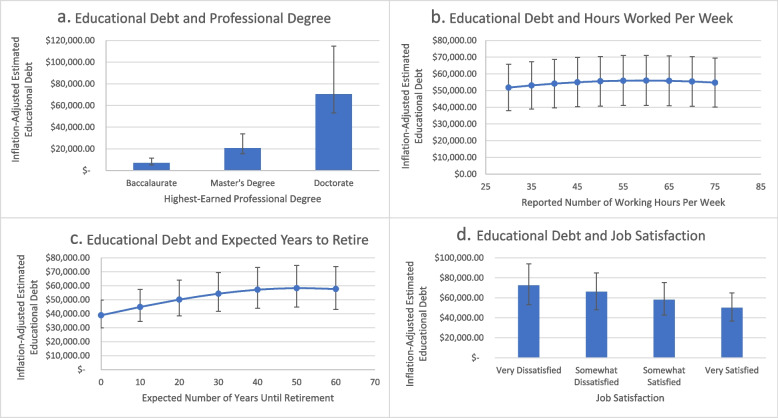
Fixed effects prediction model of the relationship between estimated profession-related educational debt and **a** highest-earned professional degree, **b** hours worked per week, **c** expected number of years until retirement, and **d** job satisfaction. Error bars represent 25^th^ and 75^th^ percentiles for the estimates

The highest-earned professional degree was our first statistically significant variable, which had a coefficient of 0.279 (*p* = 0.009) with the log-transformed variable for educational debt. Professional degree was measured in an ordinal fashion (with 1 representing a bachelor’s degree, and 3 representing the Doctor of Physical Therapy (DPT)) and because the squared professional degree term resulted in a non-linear relationship, a graphical representation of the results is most accurate, as seen in Fig. 2a. This graph includes the inverse transformation of the predicted result for educational debt for ease of interpretation. Physical therapists who earned a bachelor’s degree as their highest professional degree could expect a median debt of $7,023.38, while a physical therapist with a DPT could expect $70,403.62. This amounts to an inflation-adjusted difference of $63,380.24 between a bachelors and a DPT, and that applies to any given set of values for each of the covariates included in the model.

We theorized that those in our sample who were younger in age were more likely to have obtained the DPT degree, as the DPT was first awarded in 1995, with the full transition from a master’s degree to the DPT occurring from 1995 to 2015 [47]. Therefore, we tested for a possible interaction effect by creating a multiplicative interaction term. This interaction term was created by multiplying the (1) age, (2) professional degree, and (3) squared professional degree variables and then running the fixed-effects model again with the interaction term added as a covariate. An F-test for joint significance yielded a *p* < 0.001, and the interaction term had a coefficient of -0.017. These results indicate an interaction effect between age and the professional degree obtained on educational debt, with younger physical therapists noting a stronger relationship between professional degrees and educational debt than older physical therapists.

#### Hours worked per week

The number of hours worked per week was also positively correlated with the log-transformed variable for educational debt (coefficient of 0.004, *p* = 0.049). The results indicate that, for a one-unit difference in the number of hours worked per week, log-transformed educational debt differed by 0.004. A graphical prediction model is presented in Fig. 2b and includes the squared term for number of hours worked per week. The inverse transformation of the predicted result for educational debt is provided for ease of interpretation. Error bars represent 25th and 75th percentiles for the estimates. The graph indicates that those who worked 40 h per week were estimated to have $54,176.36 in educational debt, while those who had the habit of working an additional 10 h per week were estimated to have $55,603.42 in educational debt (a difference of $1,427.06), and that applies to any given set of values for each of the covariates included in the model.

#### Years until retirement

The expected number of years until retirement was also a statistically significant finding in the fixed effects model, with a coefficient of 0.006 (*p* = 0.013). For a one-unit difference in the expected number of years until retirement, log-transformed educational debt differed by 0.006. The graphical representation of this estimate, which includes the squared term for years until retirement, is represented in Fig. 2c and includes the inverse transformation of the predicted result for educational debt. A physical therapist who expects they will retire in 10 years is predicted to have $44,846.46 in inflation-adjusted educational debt, while a physical therapist who expects to retire in 30 years is predicted to have $54,339.14 in educational debt. This amounts to an estimated $9,492.68 of inflation-adjusted educational debt for 20 working years.

#### Job satisfaction

Job satisfaction was statistically significant and positively correlated with educational debt, with a coefficient of 0.048 (*p* = 0.042). However, when the squared interaction term for job satisfaction was entered into the fixed effects model, job satisfaction and educational debt became negatively correlated. This prediction is graphically represented in Fig. 2d and includes the inverse transformation of the predicted result for educational debt. The job satisfaction variable was ordinally ranked (1 indicating “Very Dissatisfied”, 4 indicating “Very Satisfied”). Those physical therapists who say they are very dissatisfied with their current job situation are predicted to have $72,402.78 in profession-related education debt, while those who are very satisfied are predicted to have $50,011.09 in profession-related educational debt, a difference of $22,391.69.

We theorized that age might have an interaction effect on job satisfaction. Theoretically, either burnout from the profession could cause those who are older in age to have less job satisfaction, or larger amounts of educational debt that younger physical therapists have could lead to less job satisfaction. We therefore created a multiplicative interaction term by multiplying the following 3 variables: (1) age, (2) job satisfaction, and (3) the squared job satisfaction term. This new interaction variable was then added back into the fixed effects model as an additional covariate. An F-test for joint significance was run, with *p* < 0.001, indicating that there was an interaction effect between age and job satisfaction on educational debt (coefficient = 0.005). This indicates that those who were older in age saw a stronger relationship with job satisfaction on educational debt than those who were younger in age.

To adequately assess the Labor-Search Model as a viable theory of job choice among physical therapists in our study, we felt it was necessary to test for a possible interaction effect between income and job satisfaction. A multiplicative interaction term was created by multiplying the job satisfaction variable and inflation-adjusted income. That variable was then logged and added back into the model. The F-test for joint significance, with a *p* < 0.001, indicated that there was an interaction effect between income and job satisfaction on educational debt (coefficient = -1.26). Those who had lower income noted a stronger negative relationship between their educational debt and job satisfaction.

The year surveyed was also statistically significant for both 2018 and 2020 (*p* < 0.001 in each case). This indicates that there was a difference in the amount of educational debt reported in the dataset for both 2018 and 2020, as compared with 2016. These results highlight the importance of controlling for time within a fixed effects model.

## Discussion

We found an association between educational debt and several variables, including the professional degree obtained, number of hours worked per week, expected years until retirement, and job satisfaction among physical therapists. The fixed effects model accounted for omitted variable bias and controlled for age, race and ethnicity, and year of analysis. This model, therefore, allowed us to draw more robust conclusions about the results.

### Building on our conceptual framework

The results of our study add to existing literature on the effects of educational debt on reservation wages for physical therapists, as defined in the Labor-Search Model. Higher amounts of educational debt among physical therapists appeared to be associated with higher professional degree obtainment, lower levels of job satisfaction, working more hours weekly, and a longer time horizon until retirement. Though income by itself was not a statistically significant variable, income did interact with educational debt in our model. This finding adds nuance to the Labor-Search Model [30], as those with lower levels of income had a stronger negative relationship between their educational debt and satisfaction with their current employment situation.

Descriptive statistics from our study highlight several trends that are relevant to the Labor-Search Model, such as the increasing amount of educational debt for those who are younger and who have a higher level of educational degree attainment [48, 49]. These results, while not surprising, are important for understanding the labor market. For example, those in the 25–29 age group had a median of $81,472.97 additional inflation-adjusted dollars in existing educational debt in comparison to those in the 40–44 age group, who had $0 in educational debt. While some of that difference in educational debt is that those in the 40–44 age group had more working years to pay off educational debt, a major contributor is the higher cost of education in general. The average total cost of a physical therapist program in the United States in 2000 was $18,013 for a public institution and $50,556 for a private institution [50], while in 2020 those numbers had grown to $69,418 for a public institution and $118,865 for a private institution [51].

Also contributing to the rising cost of education is the increased value of the professional degree. Additional time within the higher education system has resulted in rising tuition costs. To achieve their personal, economic, and professional goals, students are increasingly faced with higher educational debt to obtain a professional healthcare degree [15, 16]. The results of our study suggest that physical therapists are handling the cost of this degree through a higher volume of work, and lower job satisfaction is related to higher debt loads.

Of note, the number of specialty certifications a physical therapist obtained was not found to be statistically significantly correlated with profession-related educational debt. Counter to prior literature [17, 32–34, 52], our results indicate that educational debt may not be a deterrent for physical therapists in choosing to pursue a specialty certification. Based on our results, it is unclear whether some physical therapists might choose to pursue a specialty to raise their human capital or enhance their income.

### Professional degree

Physical therapists with a bachelor’s degree or master’s degree were more likely to have obtained their physical therapy degrees many years ago, as compared with the DPT, and thus would be assumed to have less educational debt. However, the model controlled for age of the respondent and adjusted educational debt to the 2022 consumer price index. Therefore, results indicate the magnitude of difference between the cost of obtaining higher degrees for this population (an inflation-adjusted difference of $63,380.24 between a bachelors and a DPT), and that applies to any given set of values for each of the covariates included in the model.

The physical therapist profession felt that the transition to a DPT was an important step in gaining the ability to serve society through autonomous physical therapist practice [47, 53]. However, the shift to a DPT may have had the unintended consequence of adding to the debt burden of new professionals. Some have cited that the advent of accelerated DPT programs provides a way to lessen this debt burden [54]. Additional ways to lower the cost of education are needed. These may include paid clinical education experiences, including having students complete year-long clinical experiences [55] in which the latter half of the experience is paid. CAPTE could consider limiting the number of credit hours a program requires for awarding the DPT. Universities could alternatively provide additional graduate assistantship options or expand programs such as Public Service Loan Forgiveness [56].

### Volume of work

Our model indicates a positive correlation between profession-related educational debt and how much physical therapists worked per week, as well as the number of years in which they expect to retire. This amounted to a difference of nearly $1,500 of inflation-adjusted debt for each additional 10 working hours per week, and that applies to any given set of values for each of the covariates included in the model. Based on these model results, students should be wary of taking out more than the necessary dollar amount to pay for their education. Though additional research is needed, it does appear that physical therapists who have higher educational debt are working more hours and with a longer time horizon, presumably to pay off their educational debt.

To assist students with addressing these issues, universities should continue to strive for additional cost transparency and return on investment. It can be difficult for prospective students to decipher the distinctions in cost between a 3-year public institution, a 2-year private hybrid program, and a 6-year Bachelor’s + DPT option. Meeting with financial planners and financial aid counselors before a program starts as well as during a program could be helpful in planning for the types and amount of student loan debt needed for cost of living [57, 58]. Prior literature suggests that education on financial management on campus can be helpful in improving students’ financial well-being [58]. Clinical education consortia should cooperate among their institutions to control costly student travel requirements during clinical education experiences. Institutions could steer students towards those post-graduation job opportunities that forgive or reduce student debt, such as those through the Indian Health Service or the Armed Services. Further, it is incumbent upon us as professionals to continue to be advocates of higher education policy that mitigates educational debt.

### Job satisfaction

We correctly predicted that those with more educational debt would have lower levels of job satisfaction. Prior literature indicates that those with higher debt may rate life satisfaction lower [18]. Increased debt also appears to have produced a similar trend with job satisfaction. This trend is concerning, as it suggests that educational debt may be a factor in burnout from the profession. This would be in keeping from prior literature on medical trainees [59, 60], emergency medicine physicians [61], and physiatrists [58]. Of note, most physical therapists in the data set reported being “Very Satisfied” with their current job situation (Table 2). This finding is in keeping with other sources, which indicate relatively high levels of job satisfaction among physical therapists, particularly those who pursue residency and fellowship training [59].

There may be greater dissatisfaction by physical therapists who have been in the workplace longer, which is supported by the observed interaction effect, which demonstrated a stronger relationship between debt and job satisfaction for those who are older in age. It is possible that the educational debt burden by the healthcare workforce takes its toll on individuals as they progress further into their career. Healthcare and educational policymakers must consider these factors, as our results suggest greater job dissatisfaction for physical therapists who have more debt at older ages. Considering the alarming rate with which educational debt is rising among newly graduating physical therapists, one would expect much poorer job satisfaction and retention within the profession in the next 20 years.

The Labor-Search Model suggests that either assistance with paying off educational debt or mitigating the effects of increasing higher education costs may improve the long-term job satisfaction, or non-wage amenities in general, for the physical therapist workforce. High educational debt among physical therapists and low job satisfaction may impact patient care. Though further research is needed, some studies have suggested an inverse relationship between patient satisfaction and burnout [62], but positive associations between burnout and medical malpractice [63].

### Limitations

Because prediction models from this study created a singular point prediction within a heterogenous sample, caution should be taken when interpreting predictions literally. What is important is the significant trend and strength of the findings regarding the increase in predicted educational debt with additional degree obtainment, increased volume of work, and lower job satisfaction.

Our data include physical therapists who renewed a license in one state from 2014 to 2020 and may therefore not be generalizable to the entire population of physical therapists in the United States. Additional research is needed in the US, in international contexts, and among other health professions to determine the generalizability of the Labor-Search Model. Further, the participants within the study may not be fully representative of physical therapists within the Commonwealth of Virginia, let alone PTs in the United States. Roughly 40% of our sample worked primarily in an outpatient practice (Hospital, Outpatient; Private Practice, Solo; and Private Practice, Group combined). A WebPT survey of 6,647 physical therapists nationwide estimated the proportion of outpatient physical therapist practitioners in the same settings to be closer to 74.4% [60], while 2017 member data from the APTA indicated that 53.1% of physical therapists worked in an outpatient setting [61].

The educational debt variable did not include debt accrued outside of profession-related activities, so it did not include debt that physical therapists may have incurred from their undergraduate education, which would likely also impact their workforce choices. We were constrained by the availability of data in the dataset. This is a limitation of our model, as the Labor-Search Model would theoretically apply to educational debt accrued from all undergraduate, graduate, and professional degrees. It is possible that the Labor-Search Model would also be predictive of the impacts of assets more broadly, including familial wealth, investments, home mortgage, and credit card debt. Further research is warranted in this area.

The use of only profession-related educational debt in the present study would explain the discrepancy between the educational debt values in our study and the reported educational debt from survey studies conducted by prior authors in the field [10, 11, 15, 16]. There is also less potential of selection bias from the present study design as compared to a survey study. Those who respond to a survey on educational debt are more likely to have educational debt, which may also help to explain the lower values of educational debt in our panel data.

The certification variable did not distinguish between APTA Specialist Certification or other profession-related certifications. It is therefore unknown whether some certifications may prove to be more of a barrier to obtain than others due to their cost. Further, our data did not include information regarding physical therapist residency or fellowship training. Because physical therapists who pursue such training can incur temporarily reduced income or higher costs, additional research should study the association between educational debt and ones’ decisions to pursue residency or fellowship training.

### Our future

The present data indicate that currently, physical therapists are generally very satisfied with their current job situation. At the same time, early career physical therapists face a high level of educational debt that falls below the value of other health professions degrees [11]. The physical therapist profession must advocate for solutions to contain the rising cost of educational debt, least the younger portion of the workforce face pressure to increase their work volumes to an unsustainable level and experience a decline in job satisfaction. Future research should focus on effective ways to mitigate educational debt in the current economic environment.

## Conclusion

Those with higher levels of profession-related educational debt tend to have the DPT degree and lower job satisfaction, but also have the work habit of working more hours per week and have a longer time horizon until retirement. Income and job satisfaction demonstrated an interaction effect on educational debt, such that those with lower levels of income had a stronger, negative relationship between their debt and job satisfaction, as compared to those with higher income. There does not appear to be an association between educational debt and the number of specialty certifications a physical therapist pursues, or the setting in which a physical therapist works. These results have important implications on the physical therapist workforce, particularly as they relate to the volume of work and job satisfaction that professions may face in the coming years.

## Data Availability

The datasets generated and analyzed for the current study are available through the Virginia Longitudinal Data System. VLDS provides authorized researchers with a secure access to data records. https://vlds.virginia.gov/insights.

## Abbreviations

APTA: American Physical Therapy Association
US: United States
VDOE: Virginia Department of Education
VDHP: Virginia Department of Health Professions
VLDS: Virginia Longitudinal Data System
